# Using mixed methods to select optimal mode of administration for a patient-reported outcome instrument for people with pressure ulcers

**DOI:** 10.1186/1471-2288-14-22

**Published:** 2014-02-12

**Authors:** Claudia Rutherford, Jane Nixon, Julia M Brown, Donna L Lamping, Stefan J Cano

**Affiliations:** 1 Clinical Trials Research Unit (CTRU), University of Leeds, Leeds, UK; 2 Department of Health Services Research and Teaching, London School of Hygiene & Tropical Medicine, London, UK; 3 Clinical Neurology Research Group, Plymouth University Peninsula School of Medicine and Dentistry, Plymouth, UK; 4 QOL Office, University of Sydney, Sydney 2006, Australia

## Abstract

**Background:**

When developing new measuring instruments or deciding upon one for research, consideration of the ‘best’ method of administration for the target population should be made. Current evidence is inconsistent in differentiating superiority of any one method in terms of quantity and quality of response. We trialed a novel mixed methods approach in early scale development to determine the best administration method for a new patient-reported outcome instrument for people with pressure ulcers (the PU-QOL).

**Methods:**

Cognitive interviews were undertaken with 35 people with pressure ulcers to determine appropriateness of a self-completed version of the PU-QOL instrument. Quantitative analysis, including Rasch analysis, was carried out on PU-QOL data from 70 patients with pressure ulcers, randomised to self-completed or interview-administered groups, to examine data quality and differential item functioning (DIF).

**Results:**

Cognitive interviews identified issues with PU-QOL self-completion. Quantitative analysis supported these findings with a large proportion of self-completed PU-QOLs returned with missing data. DIF analysis indicated administration methods did not impact the way patients from community care settings responded, supporting the equivalence of both administration versions.

**Conclusions:**

Obtaining the best possible health outcomes data requires use of appropriate methods to ensure high quality data with minimal bias. Mixed methods, with the inclusion of Rasch, provided valuable evidence to support selection of the ‘best’ administration method for people with PUs during early PRO instrument development. We consider our approach to be generic and widely applicable to other elderly or chronically ill populations or suitable for use in limited samples where recruitment to large field tests is often difficult.

## Background

High quality health outcomes research requires patient-reported outcomes (PROs) [1-3]. PRO instruments should be reliable, valid and able to detect clinical change over time [3,4]. Consideration of appropriate administration mode should also be made. Comparisons of the two main administration methods (interviewer and self-completed) have shown mixed results: higher item-response rates were found with administered methods, while others reported inconsistent effects [5]; one study found that different methods do not have a meaningful effect on repeated PRO measurements [6] while another reported biasing influences on the responses obtained [7]. Respondents are also less likely to give no answer or respond "don’t know" when self-completing [8]. A review of PRO instruments applied in older people found best completion rates following interview administration [9]. These findings are consistent with evidence suggesting completion difficulties increase with age, declining cognition and deteriorating health [10].

Determining ‘best’ administration mode for PRO instruments is key in the development process and usually tested through large scale field testing [11-13]. Ascertaining the appropriateness of different methods should take into account the: population; topic and setting; anticipated response rates; acceptability; and time available [5]. Additionally, consideration of bias from sources other than non-response, for instance, equivalence of different mode versions of the same instrument, should be made. As new psychometric methods, such as Rasch Measurement Theory (RMT) [14], are able to provide useful exploratory data in small samples (n = 30) [15], there is good potential to use these to help determine ‘best’ administration mode in early instrument development.

Pressure ulcers (PU) are a chronic wound that can occur when the skin and underlying tissue becomes damaged due to pressure or pressure in combination with shearing forces [16]. PUs are highly prevalent, a challenge to healthcare professionals, and a major problem for high-risk populations including the mobility impaired and the elderly [16,17]. Severe PUs can become a long-term chronic condition requiring extensive management and consequently reducing health-related quality of life (HRQL) [18]. Thus, assessment of PROs is particularly important and relevant in this disease area however ‘best’ methods of assessment need to be determined.

Few studies have used standardised PRO instruments with elderly people with chronic wounds [19] thus, there is little evidence pertaining to acceptability and appropriateness of administration methods for this population. Previous explorations have been conducted with general samples (e.g. mixed elderly) and the current evidence is inconsistent in differentiating superiority of any one method in quantity and quality of response; failing to support choice of administration mode. Further, people who develop PUs are largely elderly, highly dependent and/or with high levels of co-morbidity, making them a unique group.

We previously developed a PRO instrument for people with PUs (the PU-QOL instrument) intended for patient self-completion [20]. However, pretesting identified problems with item-response rates, questioning the suitability of self-completion for this patient group, particularly those aged over 70 years. This study uses a novel mixed methods approach to provide direction for the ‘best’ administration mode for the PU-QOL instrument. Specifically, we investigated differences between two administration groups to determine whether one instrument could be developed for use with both self-completed and interview-administered methods (similar responses between groups would support one version suitable for both methods) or whether two mode-specific versions were required (divergent responses would require two administration mode-specific versions).

## Methods

### Study design and sample

We investigated ‘best’ administration mode through: 1) semi-structured cognitive interviews with 35 participants with PUs to determine the appropriateness of and reasons for any difficulty with self-completion (study methods described elsewhere [20]); and 2) quantitative methods with the inclusion of RMT on PU-QOL data from patients randomised to self-completed or interview-administered groups to examine data quality and differential item functioning (DIF). We anticipated a sample of around 100 would meet the data requirement for DIF analyses [15].

Consecutive patients from 31 hospital and community National Health Services (NHS) around the UK, with existing PUs of any severity [16]. location or duration; aged over 18 years; and able to understand English were recruited between September 2009 and August 2010. Patients with only moisture lesions or who were unconscious, confused, cognitively impaired or deemed ethically inappropriate to approach (e.g. death was imminent) were excluded. To ensure equivalent clinical presentation in both administration groups, only patients able to read and write in English were included. Ethical approval was provided by a UK NHS Research Ethics Committee and all participants gave written informed consent to participation.

### Data collection procedures

To ensure the DIF analysis was a valid interpretation of group differences - in this instance, differences dependent on administration mode and not an artefact of differences within groups – through application of the eligibility criteria, participants were matched on clinical presentation and relevant underlying ability (e.g. with an existing PU; able to read and write independently) before determining equivalence of responses to scale items. Participants were then randomised to one of two groups: self-completed or interview-administered groups through a 2:1 ratio. The 2:1 ratio was used to account for the likelihood of increased missing data from self-completed PU-QOLs [20]. Randomisation was stratified by: age (≤ 70, >70 years), wound severity (superficial, severe) and healthcare setting (hospital, community).

Patients randomised to the self-complete group were provided with the PU-QOL and instructed to complete the instrument on their own. Those randomised to the interview-administered group had the PU-QOL administered to them by a tissue viability team member, following an interview user manual. Training in administering the PU-QOL was provided by one researcher (CR) to ensure standardisation across administrations.

### PU-QOL instrument

The PU-QOL version used in this study consisted of 13 scales (87-items): pain; exudate; odour; sleep; vitality; mobility; daily activities; mood; anxiety; self-consciousness and appearance; autonomy; isolation; and participation. Scales represent unique outcomes represented in a conceptual framework of HRQL specific to PUs [21]. Questions focused upon the impact of PUs on these constructs, rated by the amount of bother attributed (e.g. "During the past week, how much have you been bothered by…?") on a 4-point response scale (e.g. 0 = no bother – 3 = a lot of bother). A recall period of the past-week was chosen on clinical grounds, as changes in PU severity and symptomology often occur over days and thus a longer recall period would risk not capturing relevant impact on HRQL.

### Analyses

The qualitative analysis involved identifying dominant trends (e.g. issues occurring repeatedly) and key findings (e.g. issues reported once but considered severe). Findings were categorised by mode preference, ease of self-completion, and reasons for any difficulty. We calculated the proportion of: completed and returned PU-QOLs (response rate) and missing data (data quality) per PU-QOL and per item by mode group. A Rasch analysis was performed on each of the 13 PU-QOL scales to examine DIF [14,22,23]. The measurement properties of the PU-QOL instrument were subsequently tested in a large field test [24].

RMT provides a formal method for evaluating scale functioning against a sophisticated mathematical measurement model [25]. The Rasch model defines how a set of items should perform to generate reliable and valid measurements [26] and evaluates the legitimacy of summing items to generate those measurements [14,22]. The extent to which observed data (patients’ actual responses to scale items) are concurrent with (‘fit’) predictions of those responses from the Rasch model are examined; whereby the difference between expected and observed scores indicates the degree to which rigorous measurement is achieved [27]. The expected response structure is a probabilistic Guttman pattern, which assumes that for the same person ability, the probability of endorsing an easy item is higher than the probability of endorsing a more difficult item, and vice versa [28]. When a PRO instrument is used to discriminate between persons with different abilities, someone with higher ability is expected to affirm all items endorsed by a person with lower ability in addition to items representative of higher ability.

#### Rasch analysis: differential item functioning (DIF)

DIF analysis [29] is a technique for investigating conditional relationships between item response and group membership [30]. It is based on the assumption that respondents with similar ability (determined by total scores) should respond in similar ways to individual items regardless of gender, age or ethnicity. Groups are selected based on theoretical considerations about whether or not the construct measured is hypothesised to have the same conceptual meaning across groups. We proposed that the PU-QOL instrument’s scales should measure the same constructs - here measured HRQL specific to PUs - across administration mode groups.

DIF involves a between group analysis, indicating any patterns of responses. Using RUMM2030 [31], we examined: uniform DIF - indicated by the same amount of DIF between groups measured, regardless of person ability/disability level - and non-uniform DIF – indicated by varying magnitudes of DIF according to ability/disability level. DIF was considered at both the 1% and 5% level. Bonferroni corrections were applied to both levels to take account of multiple testing [32]. This is a method for adjusting the significance levels of individual tests when multiple tests are performed on the same data (the test-wise significance levels are divided by the number of tests) [33,34]. An exact probability value using Bonferroni adjustment is calculated in RUMM2030.

## Results

### Qualitative analysis

Qualitative findings indicated problems with PU-QOL self-completion. Despite assessed as able to self-complete, almost half the sample (43%) required assistance with completion; eight were aged ≥70 and seven <70 years (see Gorecki et al 2013 for additional results from the qualitative study [20]. Reasons for needing assistance included: i) too weak/ill; ii) unable to hold a pen; iii) visually impaired (e.g. glasses not accessible); and iv) co-morbidity (e.g. acute or chronic illness). Respondents did not read instructions, expressed difficulty selecting an appropriate response option, or left items blank rather than indicating "no bother" if: i) they had not experienced what the item referred to; ii) they experienced it but not because of PUs; or iii) it applied only in the past. These issues did not emerge when PU-QOLs were administered.

### Quantitative analysis

We screened 427 patients from 21 hospitals, 10 community services and one hospice. Eligibility was assessed for 227 (53.2%), of which 142 were eligible (62.6%); 75 (52.8%) consented to participation. Cognitive impairment and inability to self-complete were the main reason for ineligibility (47.7% and 26% respectively). Patient characteristics are presented in Table 1.

**Table 1:** Patient characteristics

	**Self-completed (n = 49)**	**Administered (n = 21)**	**Total (n = 70)**
Patient age (years)			
Mean (SD)	65 (15)	62 (16)	64 (15)
Median (range)	68 (21-85)	65 (27-93)	66 (21-93)
Under 70 years of age	25	14	39
70 years or older	24	7	31
Missing	0	0	0
Gender			
Male	33 (67.3%)	14 (66.7%)	47 (67.1%
Female	16 (32.7%)	7 (33.3%)	23 (32.9%)
Type of healthcare setting			
Acute hospital	26 (53.0%)	12 (57.1%)	38 (54.3%)
Community	23 (47.0%)	9 (42.9%)	32 (45.7%)
Pressure ulcer severity			
Superficial grades 1/2	28 (57.1%)	12 (57.1%)	40 (57.1%)
Severe grades 3/4	21 (42.9%)	9 (42.9%)	30 (42.9%)

### Response rates and data quality

Of the 75 patients recruited, 70 completed and returned PU-QOLs indicating a 93% response rate; no difference in response rate was observed by mode group. Table 2 indicates the percentage of missing data by groups: mode (self-complete and administered), age (<70 years and ≥70 years) and healthcare setting (hospital and community). For the administered group, the possible range of missed items was 0-1827 (i.e. 87 items per PU-QOL × 21 administrations = 1827 total items); a total of three PU-QOLs were returned with 29 items missed (1.6%). For the self-completed group, the possible range of missed items was 0-4263; 19 PU-QOLs were returned with 619 missed items (14.5%).

**Table 2:** Data quality – missing data

	**Self-completed (n = 49)**	**Administered (n = 21)**	Total* (n = 70)
PU-QOLs with missing data	**19** (38.8%)	**3** (14.3%)	**22** (31.4%)
Total number of PU-QOL items missed (range 1-87 items per PU-QOL)	619 (14.5%)	29 (1.6%)	648 (10.6%
Age			
Number under 70 years	(n = 12/25)^+^	(n = 2/14)^+^	(n = 14/39)^+^
Number items missed	336 (15.5%)	3 (0.3%)	345 (10.2%)
Number 70 years or older	(n = 7/24)^+^	(n = 1/7)^+^	(n = 8/31)^+^
Number items missed	283 (13.6%)	26 (4.3%)	309 (11.5%)
Type of healthcare setting			
Number acute	(n = 16/26)^+^	(n = 2/12)^+^	(n = 18/38)^+^
Number items missed	604 (26.7%)	28 (2.7%)	632 (19.1%)
Number community	(n = 3/23)^+^	(n = 1/9)^+^	(n = 4/32)^+^
Number items missed	15 (0.8%)	1 (0.1%)	16 (0.6%)

^+^Refers to the number of patients with missing data versus the total number of patients in the respective subgroup.

*A total of 70 PU-QOLs were returned and analysed (5 completed PU-QOLs were lost in the post).

Of the participants under 70 years of age who self-completed, 48% returned PU-QOLs with items missed compared to 29% of those 70 years or older that self-completed (Table 2). Of the administered group, two PU-QOLs had three items missed from those under 70 years and one PU-QOL with 26 items missed from those 70 years or older; this patient requested early completion due to feeling unwell.

A larger proportion of self-completed PU-QOLs were returned with missing data from hospitalised patients compared to those living in the community who self-completed (Table 2). Of administered PU-QOLs, two returned with 28 items missed from patients hospitalised compared to only one PU-QOL returned with one item missed from those living in the community (Table 2). A difference was observed by healthcare setting; hospitalised patients that self-completed returned PU-QOLs with the largest amount of missing data.

### Qualitative observations

PU-QOLs were examined to investigate any patterns in missing responses. The following observations were noted. Of the 19 self-completed PU-QOLs with missing data, four respondents wrote ‘n/a’ next to items missed, suggesting that the response option ‘My PU did not give me this problem’ was not used as intended. Six respondents completed only one item per scale; five missed items at random; two missed a page; one missed items from only the daily activities scale; and one mostly missed items at the beginning of the instrument. For the three administered PU-QOLs with missing data, one had one item missed; one had two items missed; and one hospital patient missed 26 items due to feeling unwell. No obvious patterns in responses emerged.

### Differential Item Functioning

**Figure 1:**
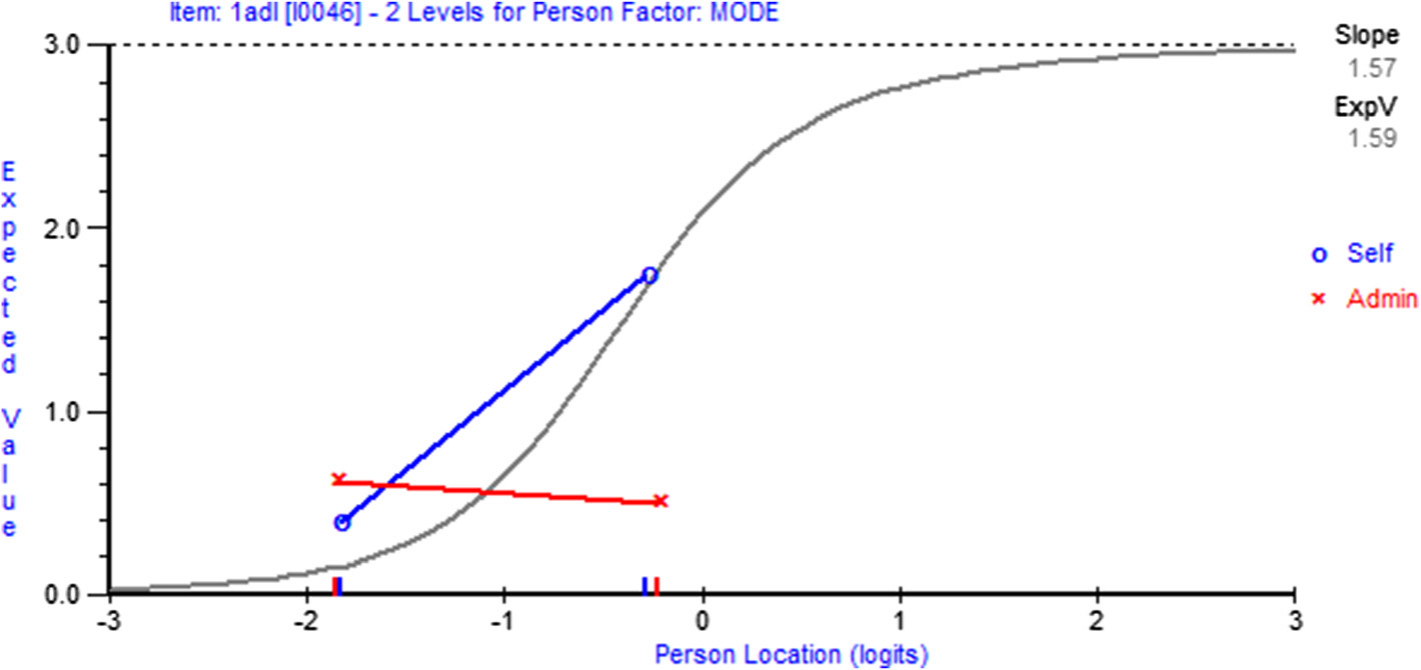
**Item characteristic curve demonstrating non-uniform item DIF.** Item characteristic curves graphically indicate the presence of item DIF. Non-uniform DIF is indicated in this item by lines on the DIF plot crossing over.

**Figure 2:**
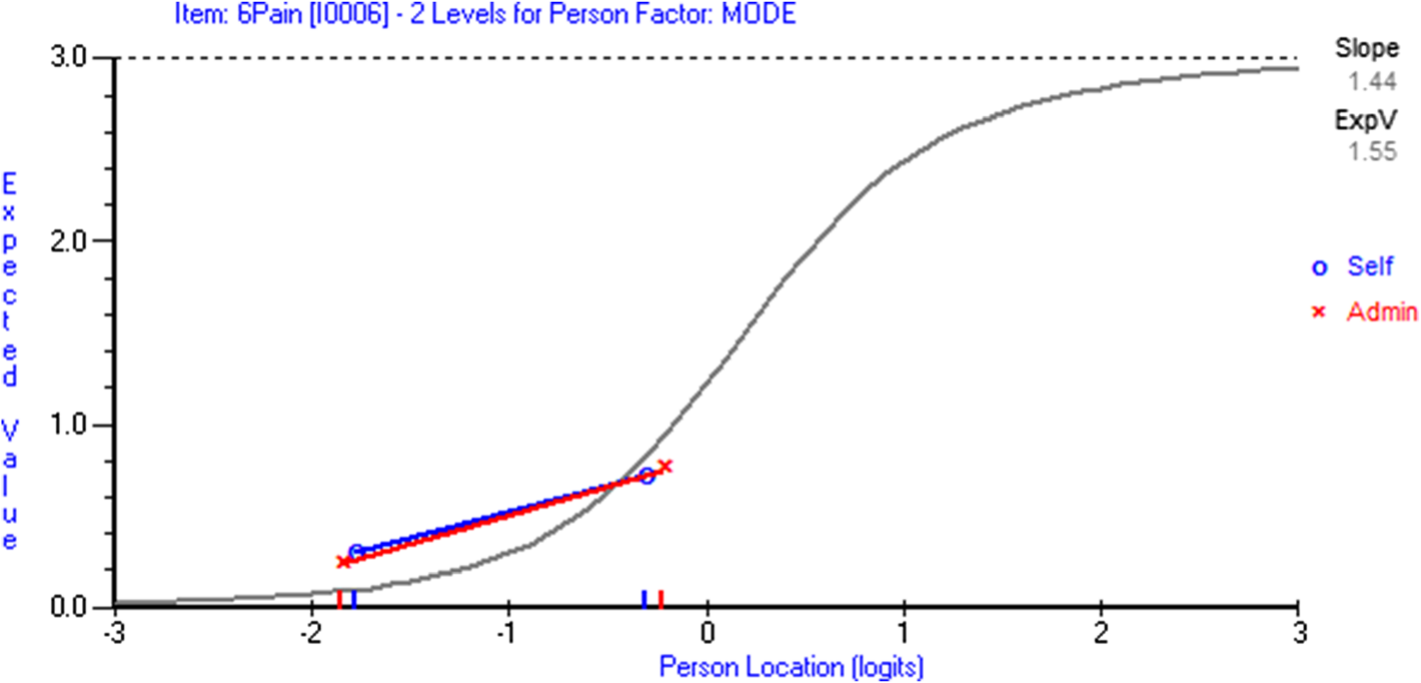
**Item characteristic curve demonstrating no item DIF.** The lines on the DIF plot run parallel and are close to each other, illustrating no item DIF. If one line was consistently higher on the DIF plot this would suggest uniform DIF in the item.

Statistically there were no items with significant DIF by mode at the 1% confidence level (Table 3); thus supporting the equivalence of self-completed and interview-administered versions. A few items emerged with DIF at the 5% confidence level; however, the DIF observed was marginal (DIF was demonstrated in 9/13 scales but only ≤3 items for seven scales; Table 3). Figures 1 and 2 provide a graphical illustration of an item with and without DIF, respectively.

**Table 3:** Summary of DIF by administration mode for each PU-QOL scale

**Scale (no. of items)**	**Uniform**		**Non-uniform**	
**(sample size)**	p < 0.01*	p < 0.05*	p < 0.01*	*p < 0.05*
**Pain (11)**				
(n = 70)	0	0	0	1
(Adjusted n = 200)	1	4	1	1
(Adjusted n = 300)	4	6	1	4
**Exudate (8)**				
(n = 70)	0	0	0	0
(Adjusted n = 200)	2	3	1	2
(Adjusted n = 300)	2	4	1	2
**Odour (6)**				
(n = 70)	0	1	0	0
(Adjusted n = 200)	3	3	3	3
(Adjusted n = 300)	3	4	3	4
**Sleep (6)**				
(n = 70)	0	0	0	1
(Adjusted n = 200)	2	3	3	3
(Adjusted n = 300)	3	4	3	3
**Malaise (3)**				
(n = 70)	0	1	0	0
(Adjusted n = 200)	2	3	1	1
(Adjusted n = 300)	2	3	1	2
**Mobility (11)**				
(n = 70)	0	1	0	3
(Adjusted n = 200)	2	3	7	7
(Adjusted n = 300)	2	4	7	9
**Daily activities (9)**				
(n = 70)	0	1	0	1
(Adjusted n = 200)	2	3	2	5
(Adjusted n = 300)	3	4	4	6
**Mood (7)**				
(n = 70)	0	0	0	1
(Adjusted n = 200)	0	2	2	4
(Adjusted n = 300)	0	4	3	5
**Anxiety (3)**				
(n = 70)	0	0	0	0
(Adjusted n = 200)	0	0	0	0
(Adjusted n = 300)	0	0	0	0
**Self-consciousness (7)**				
(n = 70)	0	0	0	0
(Adjusted n = 200)	1	4	1	2
(Adjusted n = 300)	3	4	1	3
**Autonomy (3)**				
(n = 70)	0	0	0	0
(Adjusted n = 200)	0	0	2	2
(Adjusted n = 300)	0	0	2	2
**Isolation (4)**				
(n = 70)	0	0	0	1
(Adjusted n = 200)	1	2	2	3
(Adjusted n = 300)	2	3	3	3
**Participation (9)**				
(n = 70)	0	0	0	2
(Adjusted n = 200)	4	8	7	7
(Adjusted n = 300)	6	8	7	7

*Indicates the number of items with DIF at the specified p value.

Additional exploration of DIF was undertaken with two hypothetical samples (n = 200 and n = 300); RUMM software has a function enabling multiplication of the original analysis sample (n = 70). In both adjusted samples, a significant proportion of items emerged with both uniform and non-uniform DIF (Table 3); highlighting areas warranting further investigation if pursuing a self-completed version in the future. Increasing the sample from 200 to 300 did not improve the detection of items with DIF (Table 3).

## Discussion

The PU-QOL instrument provided a vehicle for demonstrating a novel mixed methods approach to guide selection of the ‘best’ administration mode. Our findings confirm the usefulness of our strategic approach for investigating response rate, data quality and measurement equivalence between two administration methods during early PRO instrument development or in limited samples where recruitment to large field tests is often difficult.

Qualitative data informed modifications to the PU-QOL instrument. Despite modifications intended to promote self-completion, almost half the sample required assistance with completion, of which half were aged 70 years or older; findings consistent with others [35,36]. Elderly patients were more likely to miss multiple items and expressed a preference for assistance with completion. The interpersonal interaction (interviewer can provide clarification); enabling those with reading or writing difficulties to be included in research; and enhancing data quality through facilitation with visual aids or checking for data completeness makes administration of PRO instruments a suitable method for people with PUs and potentially other elderly or chronically ill populations.

A difference in data quality was observed; a large proportion of PU-QOLs that were self-completed by acute hospital patients had missing data; indicating the method was inappropriate for these patients. No difference in data quality was observed by mode for the community setting group, thus a self-completed version may be feasible for community patients; but the sample size was relatively small. Initially we had planned to include around 100 participants into this exploratory methodological study, however due to time constraints and objectives for the larger study [24], we only recruited 75 patients.

The DIF observed was marginal thus providing preliminary evidence of stable item performance across administration methods; suggesting PU-QOL scales could be measured on a common metric. However, when investigating DIF in small samples, failure to detect no DIF at the 1% confidence level does not imply that no problems exist, rather that we might not have enough power to detect measurement issues. Using the 95% confidence level indicated that the few items with DIF did not warrant two administration mode-specific versions. However, items to be cognisant of if pursuing a self-completed version in the future were identified.

Determining DIF is valuable as detection of any severely problematic items (those presenting with significant DIF) would be expected even in small samples. However, as DIF is a product of the sample and not the scale (e.g. probabilities are sample size dependent), additional exploration of DIF was undertaken. To provide confidence in our findings of marginal DIF by administration mode, we inflated the sample size to provide a better feel for the behaviour of the data and increase the likelihood of revealing any DIF [37]. Despite encouraging preliminary results, re-examination in inflated samples detected measurement non-equivalence between administration methods on some scale items. Increasing the sample from 200 to 300 did not improve detection of items with DIF, suggesting that a sample of around 200 might be required for revealing significant DIF; however optimum sample size needs to be empirically determined.

The appropriateness of different administration methods will vary depending on the population being measured, the topic and content of the scale, and the setting of the data collection. This will differ from population to population, and scale to scale, and should be empirically tested. Based on our findings, we selected interview-administered mode to ensure suitability of the PU-QOL instrument across the wide spectrum of patients with PUs and to increase clinical meaningfulness; a self-completed PU-QOL would limit the type of people that could be assessed. In longitudinal research, this can be problematic as the progress of PUs and the impact on patients may not be accurately measured due to high levels of missing responses on repeated measurement. Finally, we provide preliminary evidence for the feasibility of a community self-completed version but as this study was not powered accordingly (e.g. once the n = 33 community patients are split over the class interval groups used in the DIF analysis, a very small sample will be included in each class interval group), more work is needed to confirm appropriateness.

## Conclusion

Obtaining the best possible health outcomes data requires use of appropriate methods to ensure high quality data with minimal bias. Mixed methods, with the inclusion of RMT, provided both qualitative and empirical evidence for selection of the ‘best’ administration method for people with PUs. RMT/DIF analyses thus provide a complementary method alongside standard testing for examining key clinically reasonable variables, with the intention of flagging issues with DIF for further examination. Parallel use of qualitative methods may assist in: explaining reasons for DIF; resolving them (i.e. adapt/improve items); and testing any changes made to instruments early in the development process.

## Competing interests

The authors declare that they have no competing interests.

## Authors’ contributions

CG contributed to study concept and design, acquisition of qualitative data, analysis and interpretation of data, and preparation of manuscript. JN, JB and DL contributed to study design, interpretation of data and preparation of manuscript. SC contributed to study concept and design, analysis and interpretation of data, and preparation of manuscript.

## Pre-publication history

The pre-publication history for this paper can be accessed here:

http://www.biomedcentral.com/1471-2288/14/22/prepub
